# A multi-omics investigation into the mechanisms of hyper-virulence in *Mycobacterium tuberculosis*

**DOI:** 10.1080/21505594.2022.2087304

**Published:** 2022-07-05

**Authors:** Rahim Rajwani, Chala Galata, Annie Wing Tung Lee, Pui-Kin So, Kenneth Siu Sing Leung, Kingsley King Gee Tam, Sheeba Shehzad, Timothy Ting Leung Ng, Li Zhu, Hiu Yin Lao, Chloe Toi-Mei Chan, Jake Siu-Lun Leung, Lam-Kwong Lee, Kin Chung Wong, Wing Cheong Yam, Gilman Kit-Hang Siu

**Affiliations:** a Department of Health Technology and Informatics, Faculty of Health and Social Sciences, The Hong Kong Polytechnic University, Hong Kong Special Administrative Region, Hong Kong, China; b University Research Facility in Life Sciences, The Hong Kong Polytechnic University, Hong Kong Special Administrative Region, China; c Department of Microbiology, Li Ka Shing Faculty of Medicine, The University of Hong Kong, Hong Kong Special Administrative Region, China; d Department of Clinical Pathology, United Christian Hospital, Hong Kong Special Administrative Region, China

**Keywords:** Hypervirulence, *Mycobacterium tuberculosis*, multi-omics, tuberculous meningitis, RNA sequencing, LC-MS/MS

## Abstract

Clinical manifestations of tuberculosis range from asymptomatic infection to a life-threatening disease such as tuberculous meningitis (TBM). Recent studies showed that the spectrum of disease severity could be related to genetic diversity among clinical strains of *Mycobacterium tuberculosis (Mtb).* Certain strains are reported to preferentially invade the central nervous system, thus earning the label “hypervirulent strains”.However, specific genetic mutations that accounted for enhanced mycobacterial virulence are still unknown. We previously identified a set of 17 mutations in a hypervirulent *Mtb* strain that was from TBM patient and exhibited significantly better intracellular survivability. These mutations were also commonly shared by a cluster of globally circulating hyper-virulent strains. Here, we aimed to validate the impact of these hypervirulent-specific mutations on the dysregulation of gene networks associated with virulence in *Mtb* via multi-omic analysis. We surveyed transcriptomic and proteomic differences between the hyper-virulent and low-virulent strains using RNA-sequencing and label-free quantitative LC-MS/MS approach, respectively. We identified 25 genes consistently differentially expressed between the strains at both transcript and protein level, regardless the strains were growing in a nutrient-rich or a physiologically relevant multi-stress condition (acidic pH, limited nutrients, nitrosative stress, and hypoxia). Based on integrated genomic-transcriptomic and proteomic comparisons, the hypervirulent-specific mutations in *FadE5* (g. 295,746 C >T), *Rv0178* (p. asp150glu), *higB* (p. asp30glu), and *pip* (IS*6110*-insertion) were linked to deregulated expression of the respective genes and their functionally downstream regulons. The result validated the connections between mutations, gene expression, and mycobacterial pathogenicity, and identified new possible virulence-associated pathways in *Mtb*.

## Introduction

Tuberculosis (TB) remains a significant cause of morbidity and mortality worldwide. According to the World Health Organization global TB report, there were 10 million new TB cases and 1.4 million TB-related deaths in 2020 [1]. The globally circulating *M. tuberculosis* strains could be phylogenetically classified into seven lineages and 60 sub-lineages that share high genetic similarity (>99.98% nucleotide sequence identity) [2]. Despite limited genetic variability among them (0.02% of all nucleotide positions in the genome), remarkable strain-specific differences are observed in the region of infection (i.e. pulmonary or extra-pulmonary) [3], transmissibility [4] and virulence (i.e. degree of pathogenicity) [5]. For instance, it has been shown that strains of the *M. tuberculosis* lineage-2 (East Asian or Beijing lineage) are geographically most widespread [6], frequently associated with drug resistance [7] and hyper-virulent in animal models of TB [8]. Nevertheless, clinical strains of lineage-2 further differ in virulence [9]. Epidemiological studies have found that certain strains are able to cause disseminated disease after the lung infection and preferentially invade the central nervous system (CNS) [10,11], thus earning the label “hypervirulent strains”. It is crucial to identify the genetic factors that accounted for enhanced mycobacterial virulence in the hypervirulent *M. tuberculosis* strains.

Previously, we reported a hyper-virulent strain, H112, of the Beijing family of *M. tuberculosis*, which was isolated from the cerebrospinal fluid of an HIV-negative young patient with tuberculous meningitis. This strain also demonstrated a two-fold higher intracellular growth index and three-fold reduced TNF-α stimulation in peripheral blood derived monocyte-derived macrophages relative to 123 other strains, including the reference virulent strain *M. tuberculosis H37Rv* [12]. We compared the complete genome sequence of the hyper-virulent strain H112 with a control strain H54, which belongs to the same phylogenetic lineage (i.e. lineage 2.2.1) as H112 but was isolated from pulmonary TB patient and did not replicate in macrophages over a period of seven days [13]. Through genomic comparisons between H112 and H54 and also with previously published *M. tuberculosis* genomes in NCBI Genbank, we identified 17 mutations specific to a cluster of globally circulating hyper-virulent strains, which included H112 and other hyper-virulent strains isolated from Gran Canarias [14], Zaragoza, and Brazil [15]. The mutations affected known virulence factors of *M. tuberculosis* including *higB*-toxin (p. asp30glu), *rv3371* (p. ala323thr), *pip (IS6110*-insertion), and *fadE5* (g. 295,746 C >T) [13].

**Figure 1.**
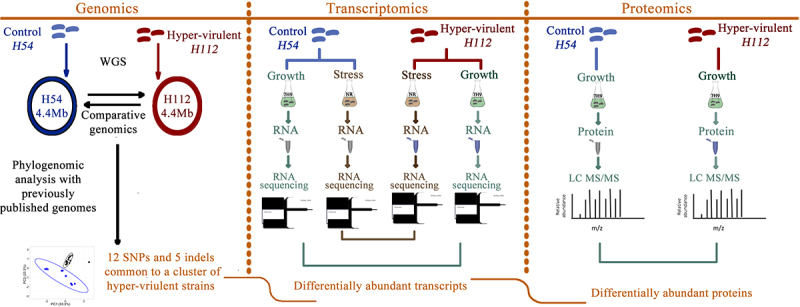
Overview of the study and experiment design.

Here, we investigated functional consequences of the hypervirulent-specific mutations by analyzing genome-wide expression differences at RNA and protein level between the hyper-virulent strain, H112, and a phylogenetically related low-virulent strain, H54, in the context of their genetic differences. We profiled the genome-wide expression pattern for each strain during exponential growth in a nutrient-rich condition (7H9 medium) and a physiologically relevant multi-stress condition (NR medium) using RNA-sequencing and label-free quantitative proteomics (Figure 1). The results uncovered that a set of genes harboring hypervirulent specific mutations were differentially expressed in strain H112 relative to strain H54 regardless of the culture condition (nutrient-rich and stress) or measurement method (RNAseq and proteomics), suggesting that these mutations are functionally important for hypervirulence by altering expression of virulence-associated genes.

## Result

### Differences in transcript abundance between hyper-virulent and low-virulent strains maintained across culture conditions

RNA extracted from each strain cultured under nutrient-rich (7H9 medium) and multi-stress condition (NR medium) was analyzed through RNA sequencing to identify differentially abundant transcripts between the hyper-virulent strain H112 and the low-virulent strain H54. The RNA sequencing runs yielded on average 33.71 million high quality (>Q30) reads, of which, 87.15% mapped uniquely to the reference genome *M. tuberculosis* H37Rv (NC_000962.3) (Fig S1). Overall, the sequencing result was consistent between replicates as indicated by close clustering of the replicates in hierarchical clustering and principal-component-analysis (PCA) (Fig S1). The clustering analysis also uncovered that the genetic mutations between the strains were important factors in shaping the transcriptional profiles of the studied strains (Fig S1).

**Figure 2.**
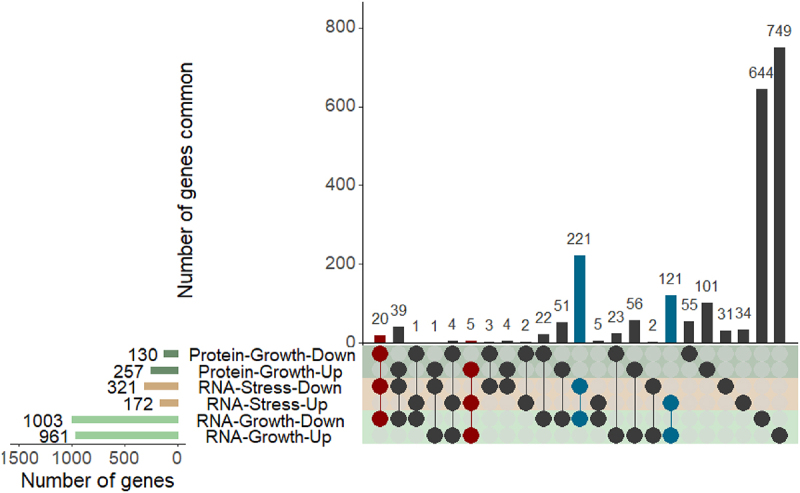
Consistent differential gene expression in H112 across 7H9 (nutrient-rich) and NR (stress) medium conditions relative to H54.

Pairwise comparison of the DESeq2 normalized read-counts per gene between strain H112 and strain H54 revealed that there were 1,964 and 493 differentially abundant transcripts (false-discovery-rate [FDR] lower than 5%) between the two strains during exponential growth in 7H9 medium and under stress in NR medium, respectively (Figure 2). To validate the RNA seq results, a set of seven genes were selected for quantification by RT-qPCR because of their association with hypervirulent-specific mutation and their considerable expression difference between H112 and H54 (fold change greater than two). The differential expression of these seven genes was confirmed to be consistent in RT-qPCR analysis (Fig S2).

The number of differentially expressed genes in each growth condition and the intersections (number of common genes) between them are illustrated. For example, in the right panel, 221 genes showed consistent differential expression (downregulated) at RNA levels across both 7H9 and NR conditions, whereas 121 genes were upregulated at RNA levels under the conditions. Consistent differential gene expression at both RNA levels across conditions is highlighted in blue. Similarly, 20 and 5 genes were downregulated and upregulated, respectively, at both RNA and protein levels (highlighted in maroon) across the conditions. The direction of expression change is indicated as in H112 relative to H54.

In addition, transcripts differentially abundant under stress-phase nearly overlapped (83.16%, 410/493) with those during the exponential growth phase, suggesting that the basal-level expression of these genes is significantly different between H112 and H54, and the gene-expression differences are maintained in NR medium, which mimics the multi-stress condition inside macrophages (Figure 2).

### Differentially abundant proteins between hyper-virulent and low-virulent strains during exponential growth in 7H9 medium

As the growth of *M. tuberculosis* strains was diminished under stress condition in NR medium, the protein yields were not sufficient for LC-MS/MS analysis. Therefore, the proteomic analysis was based on mycobacterial protein extracted from 7H9 cultures only. Whole-cell lysates from exponentially growing cultures of each strain were analyzed for their differences in protein abundance using LC-MS/MS. Clustering analysis based on principal components 1 and 2 showed minor variations between replicates from the same strain compared to samples prepared from a different strain (Fig S3), a trend that was also seen in the PCA analysis for transcriptomic data and demonstrates high technical reproducibility of both datasets (Fig S1).

A total of 3,200 peptide ions were detected across both strains which were annotated to 1,189 known proteins from reference strain *M. tuberculosis H37Rv* using Mascot algorithm (FDR < 0.01 with two or more peptides per protein) and included in the quantitative analysis. Of these 1,189 proteins, 387 were differentially abundant between H112 and H54 with log2 fold-changes ranging from −4.9 to 6.45 (*p*-value <0.05) (Figure 2). There were 257 proteins upregulated (median log2 fold-change 0.56, range 0.11 to 6.45) and 130 downregulated (median log2 fold-change −0.59, range −4.9 to −0.17) in the hyper-virulent strain H112 relative to low-virulent strain H54 (Figure 2).

### Consistent gene expression differences at RNA and protein level

**Figure 3.**
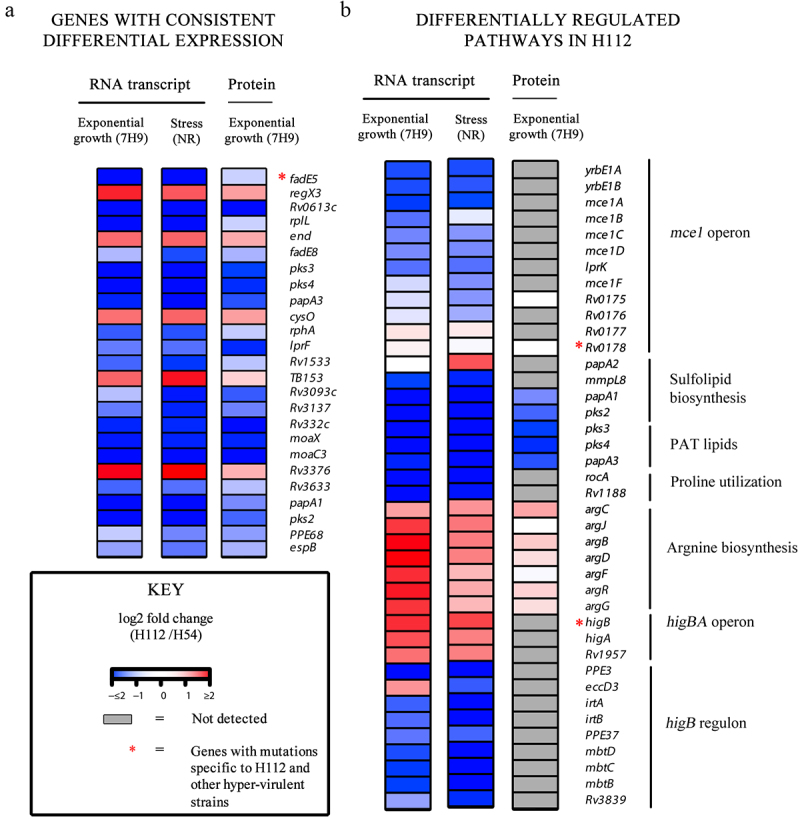
An integrated analysis of genomic, transcriptomic, and proteomic differences between hyper-virulent strain H112 and phylogenetically related low-virulent strain H54.

Twenty genes were consistently downregulated (FDR < 0.05) and five genes consistently upregulated (FDR < 0.05), regardless of the quantitation method (RNAseq or proteomics) or experimental condition (7H9 or NR) (Figure 2 and 3).

A and B depict gene expression changes in H112 relative to H54. The up- and down-regulated genes are presented with red and blue color, respectively, where the intensity of the color is proportional to the expression fold difference (p-value <0.05).

In addition, there were also 221 genes for which RNA transcripts were consistently downregulated (log2 Fold change < −1, FDR < 0.05) in both 7H9 (nutrient-rich) and NR (stress) medium in the RNAseq experiments (Figure 2), however, evidence of their differential expression at the protein level was not available, including 160 genes whose protein products were not detected in the LC-MS/MS analysis. Only two such downregulated genes encoded non-coding RNAs (*b11* and *mcr7*, log2 Fold change >1 and FDR < 0.05), whereas all others encoded proteins. A similar pattern was also noted for 121 upregulated genes (FDR < 0.05) in the RNAseq experiments, all of which encoded proteins (Figure 2).

### Differential gene expression associated with mutations in hyper-virulent strain

We previously reported 12 SNPs and 5 indels in hyper-virulent strain H112 that were shared with many other globally circulating hyper-virulent strains but absent from phylogenetically related low-virulent strain (Table 1). The following sections describe the links between these mutations and differential gene expression.

**Table 1.** Associations between genetic mutations in the hyper-virulent strain and differential gene expression.

Gene	Mutation in the hyper virulent strain (*M. tuberculosis H112* and others)		Differential transcript abundance under stress		Differential transcript abundance under exponential growth		Differential protein abundance under exponential growth	
	Nucleotide-change	Amino acid change	log2 FC	adjusted p-value^1^	log2 FC	adjusted p-value^1^	log2 FC	p-value^1^
rv0178	209387T>G	D150E	−0.053	0. 945135842	0.088	0. 66248835	0.001231521	0. 997765
rv0209	249350 G>A	A105T	−0.489	0. 49130674	−0.164	0. 523453133	ND	ND
fadE5 (Rv0244c)	295746C> T	Intergenic	−3.712	1.43E-22	−4.406	3.6477E-288	−0. 316649866	0.019496
mkl (Rv0655)	752134C>T	Synonymous	−0.473	0. 326913082	−0.718	1.12108E-07	−0.032799587	0. 894773
rv1443c (Rv1443c)	1622580C>A	R38 L	0.079	0. 933935313	−0.089	0. 75301824	−1. 15916957	0.045304
higB (Rv1955)	2201808C>G	D30E	1.256	0.006633891	1.405	2.24982E-24	ND	ND
rv2696c	3012950 G>T	A220E	−0.132	0. 845216821	−0.087	0. 655654126	0. 377485595	0. 238744
fadD29 (Rv2950c)	3301648T>G	M270 L	−1.407	0. 180054358	−1.548	5.24449E-30	1. 290589217	0.014501
agpS (Rv3107c)	3476350 G>A	Synonymous	−0.202	0. 783781504	−0.251	0. 256871347	−5.18E-01	0.001954
uvrD2 (Rv3198c)	3569220 G>A	Synonymous	−0.41	0. 442191072	−0.224	0. 189296005	ND	ND
rv3371	3785898 G>A	A323T	−2.597	2.34E-07	−2.315	1.08152E-64	ND	ND
eccC4 (Rv3447c)	3865243 G>T	A999D	−0.102	0. 938408558	0.527	0. 142335432	ND	ND
rv0633c	9bp Deletion at 730085	Within coding-sequence	0.473	0. 453204256	0.317	0.07937841	ND	ND
phoP (Rv0757)	2bp Deletion at 854259	Intergenic	1.253	0.003101432	0.844	3.74101E-11	−9.67E-02	0. 297817
pip (Rv0840c)	1358bp Insertion at 937115	Within coding-sequence	−1.257	0.004845373	−0.656	0.000120335	ND	ND
rv2168c	1bp Deletion at 2431514	Intergenic					ND	ND
rv2286c	1358bp Insertion at 2559504	Within coding-sequence	−0.445	0. 48226638	−0.852	0.000356763	ND	ND

^a^p-value <0.05 are highlighted in orange. Log2 FC refers to fold-change in H112 relative to H54. ND refers to proteins that were not detected.

#### Single-Nucleotide polymorphisms

Out of 25 genes consistently differentially expressed, *fadE5* was linked to one of the 12 previously identified SNP (*fadE5* − 113 C >T) that was specific to the cluster of highly virulent ancient lineage 2 strains (Table 1) [13]. This gene showed consistent down-regulation of RNA transcript regardless of the growth conditions (nutrient-rich medium (7H9) (log2 fold change =-4.406) and stress condition (log2 fold change = > −3.712)) of the hypervirulent *M. tuberculosis* strain H112 and the corresponding protein was also down-regulated in proteomic analysis (log2 fold change = −0. 316,649,866) as indicated in Figure 3.

Moreover, there were two hyper-virulent strain-specific SNPs (HigB p. D30E and Rv3371 p. A323T) associated with a differential transcriptional abundance (absolute log2 fold change >1 FDR < 0.05) but the corresponding proteins were not detected in proteomics analysis (Table 1).

#### Insertions/deletions (InDels)

Five genes were previously found to harbor unique indels in hypervirulent strain H112. However, the protein products of four of these disrupted genes could not be detected in both strains H112 and H54 in the proteomics dataset, although a differential transcriptional abundance was noted. These genes included indels that were shared between H112 and other hyper-virulent strains, such as *IS6110*-mediated gene disruptions in *pip* and *Rv0840c*, and a 9bp hypervirulent specific in-frame deletion in *Rv0633c* (Table 1).

### Mutations in the hyper-virulent strain linked to differential expression of downstream pathways

Functional hypervirulent strain-specific mutations (i.e. those linked with a differential expression) could also indirectly affect the expression of co-regulated genes, for instances, those in the same operon, metabolic pathway or transcription factor regulatory network. Associations between selected hypervirulent-specific mutations and expression of functionally related genes are described below:

#### Amino acid substitutions in mce1 operon: rv0178 (p. D150E)

The transcription of *yrbE1B (*encoding a membrane permease) is coupled with 12 other adjacent genes that encode an additional permease *yrbE1A*, six substrate-binding proteins (Mce1A-Mce1F), and four accessory proteins (Mam1A-Mam1E) which together constitute the *mce1* operon (*Rv0167-Rv0178*) [16,17]. In *M. tuberculosis* strain *H112*, hypervirulent-specific mutation (D150E) was harbored in *Rv0178*. No differential expression was observed for this gene at RNA and protein level. However, the transcripts of other genes within the *mce1* operon were under-expressed under both nutrient-rich (median of log2 fold changes for all genes = > −0.79) and multi-stress condition (median of log2 fold changes for all genes = > −0.72), though the corresponding protein products were not detected in the mass spectrometry analysis (Figure 3).

#### Amino acid substitutions in higB toxin: higB (p. D30E)

*HigB* is encoded within a three-gene operon (*Rv1955*- *Rv1957*) encoding the HigB toxin, cognate antitoxin HigA and a conserved hypothetical protein [18]. Under both nutrient-rich and multi-stress condition, the HigBA toxin-antitoxin system was transcriptionally overexpressed (log2 fold change >1, FDR < 0.05), although the protein for neither gene was detectable (Figure 3). At least 22 genes are previously shown to be transcriptionally repressed by the endoribonuclease toxin HigB. Here, overexpression of *higB* in H112 was associated with a low transcriptional abundance of at least nine HigB regulated genes (*PPE3*, *eccD3*, *irtA*, *irtB*, *PPE37,mbtD*, *mbtc*, *mbtB*, *Rv3839*) in H112 under both nutrient-rich and multi-stress condition (median log2 fold change >1, FDR < 0.05), however, the corresponding protein products were not detectable in proteomics experiments (Figure 3).

#### IS6110 insertion in pip

*Pip (*proline-immunopeptidase) catalyzes the release of proline residues from the peptides, which are recycled to glutamate via the proline utilization pathway (encoded within *pruAB* operon) [19]. Consistent with IS*6110*-mediated *pip* disruption in H112 (Table 1), the transcripts for *rocA-rv1188*, encoding proline utilization pathway, were also under-expressed although proteins were not detected in LC-MS/MS analysis (Figure 3). Metabolically, perturbations in proline and glutamate biosynthesis are also linked to arginine biosynthesis. The arginine biosynthesis cluster was over-expressed in hyper-virulent strain H112 under all conditions, although it did not reach statistical significance for the proteomics comparison (Figure 3).

#### Deletion of 2 base pairs in the intergenic region rv0759c-rv0760c

Our comparative whole-genome sequencing analysis identified 2 bp deletion at NC_000962.3 (854,259 – 854,261), downstream of key virulence-associated operon *phoPR* [13]. *phoP* gene is a putative transcription regulator factor of the two-component system *phoPR* in *M. tuberculosis* [20]. The deletion of the 2 bp led to the upregulation of *phoP* in the genome of hypervirulent *M. tuberculosis* strains across the 7H9 and NR growth conditions (Table 1).

## Discussion

In the current study, we described possible genetic mechanisms of hyper-virulence in a clinical strain of Beijing family of *M. tuberculosis* through genomics-transcriptomics and proteomics comparison with a phylogenetically related low-virulent strain. We observed that the hypervirulent-specific mutations, which we identified in our previous study [13], were associated with an over or under-expression of the corresponding genes in the hypervirulent strain H112 relative to phylogenetically related low-virulent strain H54. Moreover, the expression of several virulence pathways, regulated by mutated gene products, was also remarkably different between strain H112 and strain H54, providing evidence that mutations in clinical strains of *M. tuberculosis* might be related to disease severity through over or under expression of virulence pathways.

So far, most of our knowledge on mycobacterial virulence is based on laboratory-adopted reference virulent strain *M. tuberculosis H37Rv*, with a limited understanding of how genetic diversity among clinical strains affect strain virulence [5,21]. A few exploratory studies that speculated on possible mechanisms of virulence difference among clinical strains reported many virulence-associated mutations through comparisons among strains of different phylogenetic lineage using whole-genome-sequencing alone [22]. The current study presents possible mechanisms of increased virulence in clinical strains of *M. tuberculosis* through a unique integrative omics comparison between a hyper-virulent and a low-virulent clinical strain of *M. tuberculosis* that share the same phylogenetic lineage. The study integrated gene-expression differences with genetic differences and identified novel mutations associated with differential expression of virulence-associated genes and increased virulence in *M. tuberculosis*.

**Figure 4.**
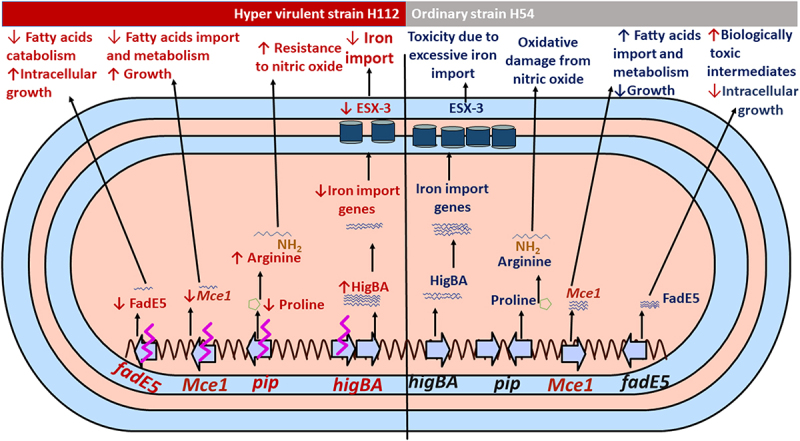
Proposed mechanisms of hyper-virulence in the clinical strain H112 compared to low-virulent strain H54.

Notably, three mutations (*rv0178* p. D150E, *higB* p. D30E and *pip* IS*6110*), which were previously identified as linked to hyper-virulence in a cluster of Beijing family of *M. tuberculosis* strains [13], were shown to be associated with significantly differential expression of the downstream gene networks under their regulation (Figure 4).

Differential expression of *higB* and *rv0178* is consistent with their previously reported role in *M. tuberculosis* virulence. For instance, Rv0178 is a component of the Mce-1 lipid transporter complex, which suppresses mycobacterial virulence and increases inflammation-mediated bacterial killing in mice [23]. The Mce-1 operon works as a transporter of fatty acids in *M. tuberculosis* and facilitates the import and metabolism of cholesterol and fatty acids through Rv3723/LucA for full bacterial virulence in vivo [24,25]. On the other hand, the strains of *M. tuberculosis* with disrupted Mce-1 operon were unable to enter a stable persistent state of infection in the lungs of mice and instead continued to replicate and killed the mice more rapidly than did the wild-type strain of *M. tuberculosis* [23]. Therefore, a possible reason for increased virulence of strain H112 could be amino acid substitution (*rv0178* p. D150E) linked to decreased transcription of all Mce-1 associated genes encoded within the same genetic locus.

Another mutation contributing to hypervirulence in H112 could be *higB* p. D30E, which is similar to *rv0178* p. D150E, is previously observed in several other hyper-virulent strains worldwide [13]. HigB *(Rv1955)* is the endoribonuclease toxin component of the tripartite toxin-antitoxin-chaperone (TAC) system HigB-HigA-SecB that cleaves mRNA for iron import genes, including the virulence-associated ESX-3 secretion system, and restricts bacterial growth in drug-induced persistence [26]. Mutation *higB* p. D30E could perturb autoregulation of the TAC system. Increased transcription of HigB-HigA-SecB operon may result in a reduced abundance of target mRNA transcripts of the genes (*PPE3*, *eccD3*, *irtA*, *irtB*, *PPE37*, *mbtD*, *mbtc*, *mbtB*, *Rv3839)* involved in iron-import, as previously reported [26] and observed in this study. The disturbance of these genes would negatively affect iron uptake pathways. While iron is essential for bacterial survival, intracellularly it must be maintained at low levels to overcome toxicity [27,28]. Thus, restricting iron import could be a mechanism of enhanced intracellular survivability of the hyper-virulent strain H112 by maintaining iron homeostasis and avoiding toxic effects.

The IS6110-mediated disruption in *pip* resulted in the transcriptional downregulation of the proline utilization pathway, which converts proline to glutamate and is encoded within the *pruAB* (*rv1187-88*) operon. Through the proline utilization pathway, the proline metabolism is also linked to arginine biosynthesis [29]. It was well reported that arginine biosynthesis plays a major role in promoting *M. tuberculosis* resistance to the interferon-ϒ-induced production of nitric oxide by macrophages. Interestingly, genes involved in the arginine biosynthesis (*argB-D*) were universally overexpressed in the hypervirulent *M. tuberculosis* H112 relative to H54. Thus, up-regulation of the arginine biosynthesis may be related to the hyper-virulence of the H112-strain to resist the nitric oxide, however, the H112 resistance to the nitric oxide was not assayed in this study and should be examined in the future.

Most importantly, in this study, *fadE5* gene demonstrated consistent down-regulation of RNA transcript and protein level in both stress and exponential growth conditions of the hypervirulent *M. tuberculosis* H112. The *fadE5* of *M. tuberculosis* plays a role in the catabolism of fatty acids, particularly in the long-chain carbon substrates used for lipogenesis in pathogenic mycobacteria [30]. The regulation of abundance and chain length of the virulence-related lipids is vital for the impermeability of the mycobacterial cell envelope because overactive fatty acids catabolism is detrimental to the pathogenicity and growth of the mycobacteria [31]. FdmR is a TetR-family transcriptional factor that was recently identified as a key regulator of *fadE5* and other genes involved in the fatty acid catabolism, and facilitates normal biosynthesis of lipid, and thus avoiding the overactive catabolism and buildup of toxic biological intermediates in the mycobacteria [31]. In the absence of the FdmR in *Mycobacterium marinum*, the bacterium was highly attenuated in both larvae and adult zebrafish, and demonstrated defective growth, but high utilization of fatty acids was noted [31], indicating how much the regulation of *fadE5* activities is important for the intracellular growth of the pathogen. In another study, the expression of *fadE5* was significantly up-regulated in *M. tuberculosis* strain *H37Rv* (fold change = 1.81) and *H37Ra* (fold change = 2.21) in intracellular environment compared to their respective growth in 7H9 broth [32], which might be the reason why the two strains (*H37Rv* and *H37Ra*) showed much lower intracellular growth and survivability in the macrophages than the hypervirulent *M. tuberculosis* H112 strain in our previous study [13]. Therefore, the downregulation of the *fadE5* gene expression in our current study could be one of the mechanisms used by the hypervirulent *M. tuberculosis* strains for maintaining fatty acids metabolism homeostasis and increases its intracellular growth and virulence in its host.

In this study, the RNA transcripts of two non-coding RNAs (b11 and mcr7) were also consistently downregulated in both 7H9 and NR conditions in the hypervirulent strains. The mcr7 gene is used as the main target for PhoP in modulating the translation of *tatC* gene and thus controlling the secretion of twin-arginine translocation substrates in *M. tuberculosis*. Therefore, it connects the PhoPR (two-component virulence system) and downstream functions essential for successful host infection [33]. The b11 modulates (reduces) the expression of several genes (*espE, panD, espF, dnaB, eccA1, mycP1, PE35,* and *MTB84*) in the *M. tuberculosis* and affects its virulence [34,35]. The expression of b11 is stimulated by low medium PH and oxidative stress, and its overexpression results in the death of *M. tuberculosis* cells [34]. In the current study, the expression of the *espF*, and *PE35 genes* was up-regulated in the hypervirulent *M. tuberculosis* strains. Hence, the down-regulation of the b11 may also contribute to the enhanced virulence of *M. tuberculosis.*

We also found that *phoP* was consistently differentially expressed in the hypervirulent *M. tuberculosis* strains across the 7H9 and NR growth conditions. The enhanced *phoP* potentially repressed at least 14 genes, including *pks2*, *pks3* that are responsible for the biosynthesis of acyltrehalose lipids, such as sulfolipids and polyacyltrehalose lipids (Figure 3). It was proposed that the loss-of-sulfolipid biosynthesis confers an advantage to the intracellular growth of *M. tuberculosis* in macrophages [36]. Our previous study also confirmed that hypervirulent *M. tuberculosis* strains with enhanced *phoP* expression displayed a significantly higher intracellular growth and survivability in macrophages [13]. Therefore, we believe that the mycobacterium infects macrophages and uses them as a carrier – like a Trojan Horse – to cross the blood–brain barrier (BBB) and establish infection in the CNS [37]. Thus, the observed expression of *phoP* indicates that hypervirulence may enable *M. tuberculosis* in causing infection in the CNS.

The current study was limited in a few aspects. Although a highly sensitive mass spectrometry instrument was used for quantitative comparison of protein abundance, only 30% of known *M. tuberculosis* proteins were detected in the LC-MS/MS analysis, owing to the limitations of current data-dependent acquisition methods in proteomics [38], which made it infeasible to confirm protein expression difference between the two strains for many protein-coding genes in *M. tuberculosis*. Nevertheless, accurate and reproducible measurements of gene expression differences were ensured by recording transcript abundance in duplicate under two experimental conditions using RNA sequencing and results were confirmed using qRT-PCR. Another limitation of the study is that experiments were not conducted to determine a definitive causative role of described mutations in modulating gene expression and virulence. Associations described in the current study should therefore be validated in the future by replacing mutant alleles in the hyper-virulent strain with wild types using techniques of genetic recombination [39]. Gene expression and virulence of the recombinant strain should be subsequently compared to hyper-virulent strain, as described in previous [13] and the current studies, where a change might indicate the contribution of the mutant allele in modulating gene expression and virulence.

## Conclusions

Using an integrated genomics-transcriptomics and proteomics approach, the study identified novel associations between genetic mutations and differential expression of virulence-associated genes and increased virulence in a clinical strain of *M. tuberculosis*. This improves our fundamental understanding of the genotype–phenotype relationship in *M. tuberculosis*, which is much needed to develop new drugs and vaccines against TB [5,21]. The results indicated that mutations specific to a cluster of hyper-virulent *M. tuberculosis* strains [13] might be related to increased virulence by modulating the expression of virulence-associated pathways, an important lead that needs to be validated further in the future studies using techniques of genetic recombination in *M. tuberculosis*.

## Materials and methods

### Bacterial strains

The experiments with viable *M. tuberculosis* were performed in a biosafety level 3 laboratory. The study was ethically approved by the Institutional Review Board of the Hong Kong Polytechnic University (Ref. number: RSA15096). A hypervirulent clinical strain *M. tuberculosis* H112 and a low-virulent strain *M. tuberculosis* H54 that share the same phylogenetic lineage were selected in this study. The virulence and phylogeny of these strains have been described in previous studies [12,13]. Briefly, *M. tuberculosis* H112 was isolated from an HIV-negative tuberculous meningitis patient in Hong Kong. *M. tuberculosis* H112 demonstrated enhanced intracellular survival in macrophages relative to 123 clinical strains. On the other hand, *M. tuberculosis* H54 demonstrated limited survival in macrophages and was isolated from pulmonary tuberculosis patient. Both H112 and H54 are members of *M. tuberculosis* lineage 2.2.1.

### Medium and culture conditions

For each *M. tuberculosis* strain, duplicate cultures were grown to log phase in Middlebrook 7H9 broth supplemented with 10% oleic-albumin-dextrose-catalase (OADC) growth supplement, 0.2% glycerol, and 0.02% tyloxapol. RNA was isolated from one of the cultures, while the other was pelleted down, washed with phosphate-buffered-saline (PBS), and resuspended in a multi-stress non-replicating (NR) medium with simultaneous exposure to multiple physiological stresses, including acidic pH, limited nutrients, nitrosative stress, and hypoxia, which mimic the growth condition of *M. tuberculosis* during macrophage challenge. The composition of the NR medium is detailed in previous study [40]. Briefly, it comprised of a modified Sauton’s base medium (0.5 g/L potassium dihydrogen phosphate, 0.5 g/L magnesium sulfate, and 0.05 g/L ammonium citrate) with 10% albumin-NaCl, 0.02% tyloxapol, 0.0001% zinc sulfate, 0.1% ammonium chloride, 0.05% butyrate, and 0.5 mM sodium nitrite with pH adjusted to 5.0. The cells were incubated in the NR medium for 72 h and processed for RNA isolation subsequently. The entire experiment was repeated twice.

### Comparative genomics

The details of the comparative genomics between H112 and H54 have been described previously [13]. Briefly, the whole-genome-sequence of both strains was determined using PacBio sequencing technology and de novo assembled into a single contig. A multiple core-genome alignment was constructed using complete genomes of H112, H54 and 20 other genomes representative of all major lineages of *M. tuberculosis*. SNPs and insertions/deletions specific to H112 and H54 were extracted based on the multiple-genome-alignment and annotated with respect to the reference genome *M. tuberculosis* H37Rv (NC_000962.3). The complete genome sequence of H112 and H54 are available from the NCBI BioProject accession PRJNA369711.

### RNA sequencing

#### RNA isolation

Before RNA isolation, the cultures were treated with Bacteria RNA protect reagent (Qiagen, USA) to stabilize the gene expression profile. The cells were lysed by treatment with lysozyme (100 mg/ml), proteinase k (20 mg/mL), followed by mechanical lysis using Tissue Lyser II (Qiagen). The total RNA was purified from the cell lysates with RNeasy Mini Kit (Qiagen, USA) according to the manufacturer’s instructions. The isolated RNA was treated twice with RNase-free DNase (Qiagen, USA) to remove any residual genomic DNA. As a quality control step, the integrity of the RNA was assessed using Agilent RNA 6000 Nano kit (Agilent Genomics, USA). The 23S/16S ratio was equal to 1 and RNA integrity number (RIN) was greater than 7.0.

#### Library preparation and RNA-sequencing

A total of 5 μg RNA was treated with DNase I (New England BioLabs, USA). The total RNA was depleted of the ribosomal-RNAs (rRNA) using Ribo-Zero-Magnetic-Kit (epicenter). The rRNA-depleted RNA was fragmented into 130–170 nucleotides and transcribed into cDNA using Superscript II (Invitrogen, USA). The cDNA was then poly-adenylated and ligated to the sequencing adapters. The resulting libraries were quantitated by real-time quantitative PCR (qPCR) and amplified on the cBot for cluster generation (TruSeq PE Cluster Kit v3-cBot-HS). The libraries were sequenced paired-end (90nt × 20) on a HiSeq-2000 machine (Illumina).

#### Differential gene expression analysis

Raw RNA-sequencing reads with a Phred-scaled quality score greater than 20 were aligned to the reference genome *M. tuberculosis* H37Rv (NC_000962.3) using Bowtie 2 [41]. The number of reads mapped to annotated features (n = 4,008) in the reference NCBI GenBank assembly GCF_000195955.2 was counted using feature-count. The read counts were normalized using DESeq2 [42]. Fold-changes with a false-discovery rate (FDR) less than 0.05 were considered significantly differentially expressed.

### Label-Free quantitative proteomics

#### Protein extraction

A total of 30 mL of bacterial cultures were grown to logarithmic phase in Middlebrook 7H9 broth (Becton Dickinson, USA) supplemented with 10% (v/v) Oleic acid-albumin-dextrose- catalase complex (Becton Dickinson, USA), and 0.05% (v/v) tween-80. The bacterial cells were centrifuged (5,000 g, 10 min) and the culture medium was discarded. The cell pellet was re-suspended into 1 mL of protease inhibitor cocktail (Roche, Germany) and transferred to a VK01 tube (Bertin Technologies, France) for mechanical lysis. The bacterial cells were lysed by beat beating at maximum speed for 5 min in a Tissue lyser II (Qiagen, USA). The cell lysate was centrifuged (14,000 g, 15 min) and the supernatant was transferred to a clean tube for protein extraction.

A total of 100 µL of the bacterial cell lysate was processed for the methanol-chloroform (4:1) protein extraction method as described previously [43]. The extracted protein amount was quantified using Bradford protein assay (Bio-Rad, USA). 10 µg of protein from each sample was reduced with 100 mM Dithiothreitol (Sigma, USA), alkylated with 400 mM Iodoacetamide (Sigma, USA) and digested with sequencing-grade trypsin (Promega, USA) at a ratio of 1:40 (trypsin: protein) overnight at 37°C. The digested peptides were desalted on a pierce C18 spin column (Thermofisher Scientific, USA) and dried in a speed vac. The samples were resuspended in 0.1% (v/v) formic acid and subjected to mass-spectrometry analysis.

#### Data acquisition

The MS/MS spectra was acquired in a positive ion mode in a data-dependent manner on an Orbitrap Fusion Lumos Mass Spectrometer (Thermofisher Scientific, USA) coupled to a liquid chromatography instrument UltiMate 3000 RSLCnano (Thermofisher Scientific, USA). The liquid chromatography instrument was equipped with a reverse phase C18 column for fractionation of the peptides before being analyzed in the mass spectrometry instrument. In the mass spectrometry instrument, the MS/MS analysis was conducted through a survey MS1 scan in the mass range 400 to 1,400 *m/z*, followed by the selection of the 10 most abundant ions for MS/MS fragmentation analysis. The fragmentation of precursor ions was achieved through collision-induced dissociation (CID). For each parent ion, the maximum signal accumulation time was 100 ms before being dynamically excluded for 12 secs and reconsidered.

#### Data analysis

Raw spectra were imported into the Progenesis QI software (Nonlinear dynamics, UK). For normalization and peak calling, one of the samples was selected as the reference and all other samples were aligned against it. A list of peptide ions with significantly different normalized peak intensities between the two groups was obtained, which was then annotated through peptide searches against the reference proteome of *Mycobacterium tuberculosis H37Rv* (NC_000962.3) using the Mascot server (Matrix Science, UK). The Mascot parameters for peptide searches were set as up to one missed trypsin cleavage, fixed post-translational carbamideomethyl modification of the cysteine residues, variable oxidation of the methionine, +2 or + 3 charges of the peptide ions and up to 0.6 Daltons mass error tolerance. The false discovery rate (FDR) was computed by setting a decoy database. Peptide identifications with FDR greater than 1% were excluded. The Mascot annotations were imported into the Progenesis QI software and proteins with differentially abundant expression (adjusted *p*-value <0.05) between the two groups were identified. Proteins were only considered differentially abundant if more than one peptide led to the significant difference.

### Quantitative reverse-transcriptase polymerase-chain-reaction

For qPCR validation of RNA seq results, a set of seven genes were selected for their association with an H112-specific mutation and for their considerable expression difference between H112 and H54 (fold change greater than two) to be reliably quantified using qPCR assays. For an accurate qPCR-based gene expression analysis, all PCR primers were designed to match PCR amplification conditions with *rrs* (Table S1), the housekeeping gene used to normalize the cycle threshold values. The primers used for qPCR are listed in Table S1. A two-step qPCR strategy was used with 100 ng of total RNA as the starting material. The complementary DNA (cDNA) synthesis was primed with 50 μM random hexamers (New England Bio Labs, USA) and 10 mM of each dNTP was also added to this mixture. The RNA-primer mix was heated at 65°C and cooled on ice for 1 min. The reverse-transcription (RT) reaction was setup in a 20 µL volume with 200 units of Superscript IV (Invitrogen, USA), 100 mM Dithiothreitol, and 13 μL of RNA primer-mix. The RT reaction was incubated at 55°C for 10 min and inactivated by heating at 80°C for 10 min in a Veriti Thermal Cycler (Applied Biosystems, USA).

Subsequently, singleplex qPCR reactions were set up in a 96-well plate. The PCR reaction contained 0.3 µM of each primer (forward and reverse), 5 µL of cDNA, and 25 µL Universal SYBR Green master mix (Roche) in a total volume of 50 µL. The PCR reactions were transferred to Roche LightCycler480 machine with cycling conditions set as follows: an initial enzyme activation of 10 min at 95°C followed by 40 cycles of 15 sec denaturation at 95°C and real-time quantification for 1 min at 60°C The cycle threshold (Ct) values were normalized to *rrs* and fold-changes were calculated using the 2^−ΔΔCT^ method as described previously [44].

## Supplementary Material

Supplemental Material

## Data Availability

The raw data of RNA seq and LC-MS/MS and the expression differences for each gene between hyper-virulent strain H112 and ordinary virulent strain H54 can be accessed through https://www.ncbi.nlm.nih.gov/geo/query/acc.cgi?acc=GSE203662 (RNA) and https://doi.org/10.1080/21505594.2022.2087304 (Protein).

## References

[1] World_Health_Organization. Global tuberculosis report 2020. Geneva: World Health Organization; 2020. (Licence; CC BY-NC-SA 3.0 IGO).

[2] Coll F, McNerney R, Guerra-Assuncao JA, et al. A robust SNP barcode for typing Mycobacterium tuberculosis complex strains. Nat Commun. 2014;5(1):4812. 25176035 10.1038/ncomms5812 PMC4166679

[3] Click ES, Moonan PK, Winston CA, et al. Relationship between Mycobacterium tuberculosis phylogenetic lineage and clinical site of tuberculosis. Clin Infect Dis. 2012;54(2):211–219. 22198989 10.1093/cid/cir788

[4] Aguilar D, Hanekom M, Mata D, et al. Mycobacterium tuberculosis strains with the Beijing genotype demonstrate variability in virulence associated with transmission. Tuberculosis (Edinb). 2010;90(5):319–325. 20832364 10.1016/j.tube.2010.08.004

[5] Coscolla M, Gagneux S. Consequences of genomic diversity in Mycobacterium tuberculosis. Semin Immunol. 2014;26(6):431–444. 25453224 10.1016/j.smim.2014.09.012 PMC4314449

[6] Merker M, Blin C, Mona S, et al. Evolutionary history and global spread of the Mycobacterium tuberculosis Beijing lineage. Nat Genet. 2015;47:242–249. 25599400 10.1038/ng.3195 PMC11044984

[7] Lasunskaia E, Ribeiro SC, Manicheva O, et al. Emerging multidrug resistant Mycobacterium tuberculosis strains of the Beijing genotype circulating in Russia express a pattern of biological properties associated with enhanced virulence. Microbes Infect. 2010;12(6):467–475. 20215000 10.1016/j.micinf.2010.02.008

[8] Tsenova L, Harbacheuski R, Sung N, et al. BCG vaccination confers poor protection against M. tuberculosis HN878-induced central nervous system disease. Vaccine. 2007;25(28):5126–5132. 17241704 10.1016/j.vaccine.2006.11.024 PMC1994581

[9] Kato-Maeda M, Shanley CA, Ackart D, et al. Beijing sublineages of Mycobacterium tuberculosis differ in pathogenicity in the guinea pig. Clin Vaccine Immunol. 2012;19(8):1227–1237. 22718126 10.1128/CVI.00250-12 PMC3416080

[10] Arvanitakis Z, Long R, Hershfield E, et al. M. tuberculosis molecular variation in CNS infection: evidence for strain‐dependent neurovirulence. Neurology. 1998;50(6):1827–1832. 9633735 10.1212/wnl.50.6.1827

[11] Caws M, Thwaites G, Dunstan S, et al. The influence of host and bacterial genotype on the development of disseminated disease with Mycobacterium tuberculosis. PLoS Pathog. 2008;4(3):e1000034. 18369480 10.1371/journal.ppat.1000034 PMC2268004

[12] Wong KC, Leong WM, Law HK, et al. Molecular characterization of clinical isolates of Mycobacterium tuberculosis and their association with phenotypic virulence in human macrophages. Clin Vaccine Immunol. 2007;14(10):1279–1284. 17715326 10.1128/CVI.00190-07 PMC2168117

[13] Rajwani R, Yam WC, and Zhang Y, et al. Comparative whole-genomic analysis of an ancient L2 lineage Mycobacterium tuberculosis reveals a novel phylogenetic clade and common genetic determinants of hypervirulent strains. Front Cell Infect Microbiol. 2018;7:539. 29376038 10.3389/fcimb.2017.00539 PMC5770396

[14] Alonso H, Aguilo JI, Samper S, et al. Deciphering the role of IS6110 in a highly transmissible Mycobacterium tuberculosis Beijing strain, GC1237. Tuberculosis (Edinb). 2011;91(2):117–126. 21256084 10.1016/j.tube.2010.12.007

[15] Ribeiro SC, Gomes LL, Amaral EP, et al. Mycobacterium tuberculosis strains of the modern sublineage of the Beijing family are more likely to display increased virulence than strains of the ancient sublineage. J Clin Microbiol. 2014;52(7):2615–2624. 24829250 10.1128/JCM.00498-14 PMC4097719

[16] Casali N, White AM, Riley LW. Regulation of the Mycobacterium tuberculosis mce1 operon. J Bacteriol. 2006;188(2):441–449. 16385033 10.1128/JB.188.2.441-449.2006 PMC1347267

[17] Uchida Y, Casali N, White A, et al. Accelerated immunopathological response of mice infected with Mycobacterium tuberculosis disrupted in the mce1 operon negative transcriptional regulator. Cell Microbiol. 2007;9:1275–1283. 17223927 10.1111/j.1462-5822.2006.00870.x

[18] Fivian-Hughes AS, Davis EO. Analyzing the regulatory role of the HigA antitoxin within Mycobacterium tuberculosis. J Bacteriol. 2010;192(17):4348–4356. 20585061 10.1128/JB.00454-10 PMC2937366

[19] Liu L-K, Becker DF, Tanner JJ. Structure, function, and mechanism of proline utilization a (PutA). Arch Biochem Biophys. 2017;632:142–157. 28712849 10.1016/j.abb.2017.07.005 PMC5650515

[20] Pérez E, Samper S, Bordas Y, et al. An essential role for phoP in Mycobacterium tuberculosis virulence. Mol Microbiol. 2001;41(1):179–187. 11454210 10.1046/j.1365-2958.2001.02500.x

[21] Coscolla M, Gagneux S. Does M. tuberculosis genomic diversity explain disease diversity? Drug Discov Today Dis Mech. 2010;7(1):e43–e59. 21076640 10.1016/j.ddmec.2010.09.004 PMC2976975

[22] Jia X, Yang L, Dong M, et al. The bioinformatics analysis of comparative genomics of Mycobacterium tuberculosis complex (MTBC) provides insight into dissimilarities between intraspecific groups differing in host association, virulence, and epitope diversity. Front Cell Infect Microbiol. 2017;7. DOI:10.3389/fcimb.2017.00088 PMC5360109 28377903

[23] Shimono N, Morici L, Casali N, et al. Hypervirulent mutant of Mycobacterium tuberculosis resulting from disruption of the mce1 operon. Proc Natl Acad Sci U S a. 2003;100(26):15918–15923. 14663145 10.1073/pnas.2433882100 PMC307668

[24] Nazarova EV, Montague CR, and Huang L, et al. The genetic requirements of fatty acid import by Mycobacterium tuberculosis within macrophages. Elife. 2019;8:e43621. 30735132 10.7554/eLife.43621 PMC6368401

[25] Nazarova EV, Montague CR, La T, et al. Rv3723/luca coordinates fatty acid and cholesterol uptake in Mycobacterium tuberculosis. Elife. 2017;6:e26969. 28708968 10.7554/eLife.26969 PMC5487216

[26] Schuessler DL, Cortes T, Fivian‐hughes AS, et al. Induced ectopic expression of HigB toxin in M ycobacterium tuberculosis results in growth inhibition, reduced abundance of a subset of mRnas and cleavage of tmRNA. Mol Microbiol. 2013;90(1):195–207. 23927792 10.1111/mmi.12358 PMC3912914

[27] Pandey R, Rodriguez GM. IdeR is required for iron homeostasis and virulence in Mycobacterium tuberculosis. Mol Microbiol. 2014;91(1):98–109. 24205844 10.1111/mmi.12441 PMC3902104

[28] Sritharan M, Margolin W. Iron homeostasis in Mycobacterium tuberculosis: mechanistic insights into siderophore-mediated iron uptake. J Bacteriol. 2016;198(18):2399–2409. 27402628 10.1128/JB.00359-16 PMC4999934

[29] Kanehisa M, Goto S. KEGG: Kyoto encyclopedia of genes and genomes. Nucleic Acids Res. 2000;28(1):27–30. 10592173 10.1093/nar/28.1.27 PMC102409

[30] Chen X, Chen J, Yan B, et al. 2020. Structural basis for the broad substrate specificity of two acyl-CoA dehydrogenases FadE5 from mycobacteria. Proceedings of the National Academy of Sciences. 117:16324–16332. 10.1073/pnas.2002835117 PMC7368279 32601219

[31] Dong W, Nie X, Zhu H, et al. 2021. Mycobacterial fatty acid catabolism is repressed by FdmR to sustain lipogenesis and virulence. Proceedings of the National Academy of Sciences of the United States of America. 118:e2019305118. 33853942 10.1073/pnas.2019305118 PMC8072231

[32] Li AH, Waddell SJ, Hinds J, et al. Contrasting transcriptional responses of a virulent and an attenuated strain of Mycobacterium tuberculosis infecting macrophages. PLoS One. 2010;5(6):e11066. 20548782 10.1371/journal.pone.0011066 PMC2883559

[33] Solans L, Gonzalo-Asensio J, Sala C, et al. The PhoP-dependent ncRNA mcr7 modulates the TAT secretion system in Mycobacterium tuberculosis. PLoS Pathog. 2014;10(5):e1004183. 24874799 10.1371/journal.ppat.1004183 PMC4038636

[34] Arnvig KB, Young DB. Identification of small RNAs in Mycobacterium tuberculosis. Mol Microbiol. 2009;73(3):397–408. 19555452 10.1111/j.1365-2958.2009.06777.x PMC2764107

[35] Ostrik AA, Azhikina TL, Salina EG. Small noncoding RNAs and their role in the pathogenesis of Mycobacterium tuberculosis Infection. Biochem Biokhimiia. 2021;86(S1):S109–S119. 10.1134/S000629792114008X PMC7905965 33827403

[36] Gilmore SA, Schelle MW, Holsclaw CM, et al. Sulfolipid-1 biosynthesis restricts Mycobacterium tuberculosis growth in human macrophages. ACS Chem Biol. 2012;7(5):863–870. 22360425 10.1021/cb200311s PMC3355658

[37] van Leeuwen LM, Boot M, Kuijl C, et al. Mycobacteria employ two different mechanisms to cross the blood-brain barrier. Cell Microbiol. 2018;20:e12858. 29749044 10.1111/cmi.12858 PMC6175424

[38] Sidoli S, Kulej K, Garcia BA. Why proteomics is not the new genomics and the future of mass spectrometry in cell biology. The Journal of cell biology. 2017;216:21–24. 27956468 10.1083/jcb.201612010 PMC5223617

[39] Gopinath K, Warner DF, and Mizrahi V. Targeted gene knockout and essentiality testing by homologous recombination. Mycobacteria Protocols. 2015;1285 :131–149. 10.1007/978-1-4939-2450-9_8 25779314

[40] Gold B, Warrier T, and Nathan C. A multi-stress model for high throughput screening against non-replicating Mycobacterium tuberculosis. Mycobacteria Protocols. 2015;1285:293–315. 10.1007/978-1-4939-2450-9_18 25779324

[41] Langmead B, Salzberg SL. Fast gapped-read alignment with Bowtie 2. Nat Methods. 2012;9(4):357–359. 22388286 10.1038/nmeth.1923 PMC3322381

[42] Love MI, Huber W, Anders S. Moderated estimation of fold change and dispersion for RNA-seq data with DESeq2. Genome Biol. 2014;15(12):550. 25516281 10.1186/s13059-014-0550-8 PMC4302049

[43] Wessel D, Flugge UI. A method for the quantitative recovery of protein in dilute solution in the presence of detergents and lipids. Anal Biochem. 1984;138(1):141–143. 6731838 10.1016/0003-2697(84)90782-6

[44] Siu GKH, Yam WC, Zhang Y, et al. An upstream truncation of the furA-katG operon confers high-level isoniazid resistance in a Mycobacterium tuberculosis clinical isolate with no known resistance-associated mutations. Antimicrob Agents Chemother. 2014;58(10):6093–6100. 25092698 10.1128/AAC.03277-14 PMC4187958

