# Supplementary material for: A multi-omics investigation into the mechanisms of hyper-virulence in Mycobacterium tuberculosis
Source: Virulence. 2022 Jul 5;13(1):1088–100. doi: 10.1080/21505594.2022.2087304 (PMC9262360; doi:10.1080/21505594.2022.2087304)
Supplement: Supplemental Material [file KVIR_A_2087304_SM3091.docx]

Supplementary figures and tables

# Supplementary figures


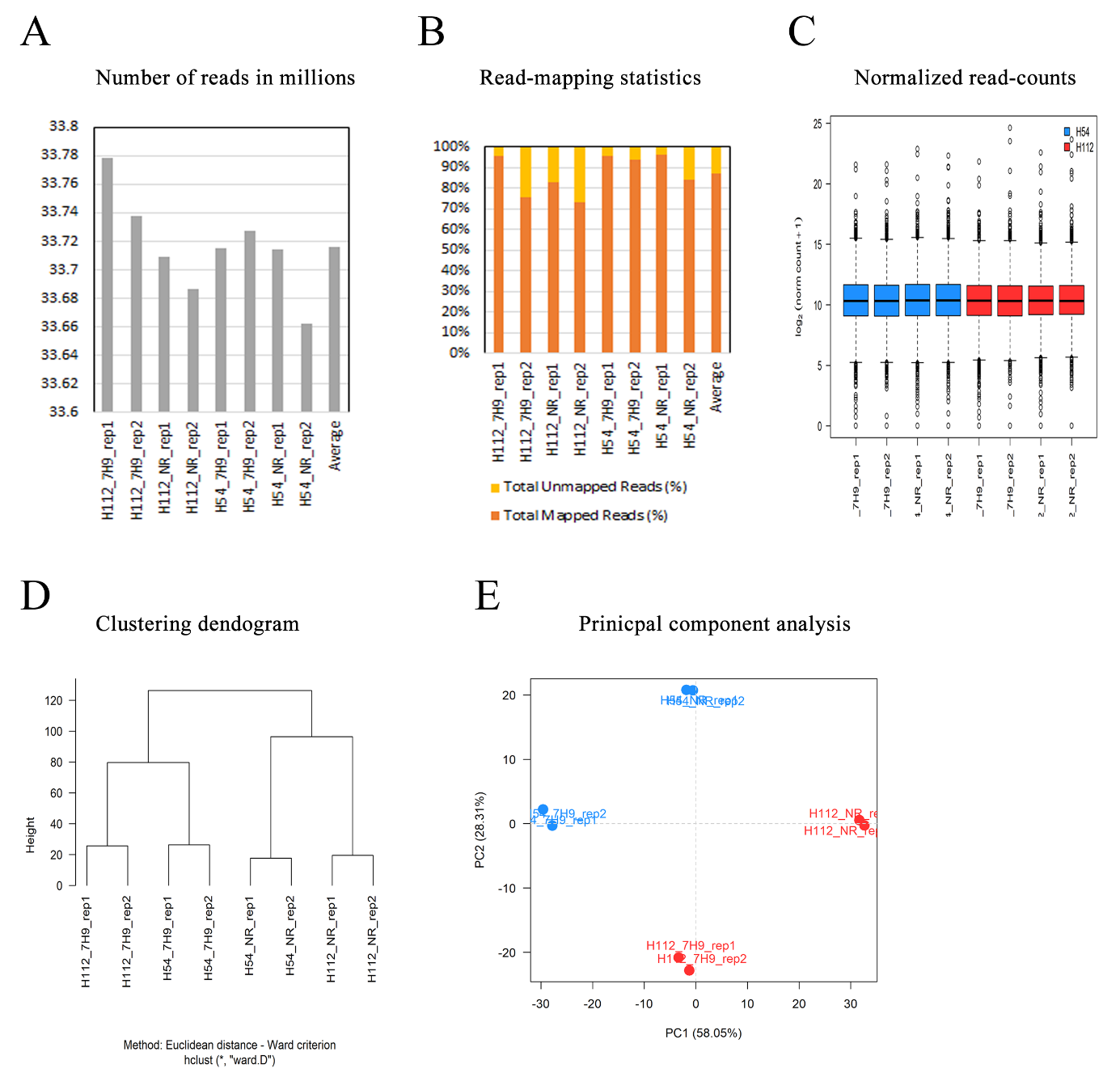


Figure S 1: Sequencing metrics for the RNA-sequencing data

Descriptive characteristics for all eight RNA sequencing data are shown. A) The number of quality-filtered reads (average base-quality above 20 after removing technical sequences) obtained for each sequenced sample is presented in a bar-plot. B) Percentage of the quality-filtered reads that mapped to the reference genome M. tuberculosis H37Rv (NC_000962.3) in each sample. C) The distribution of the gene-wise read-counts post-normalization. D) A hierarchical clustering dendrogram based on Euclidean distance matrix computed from variance-stabilizing-transformed (VST) gene-wise read-counts. E) Principal-component-analysis axes 1 and 2 are shown.


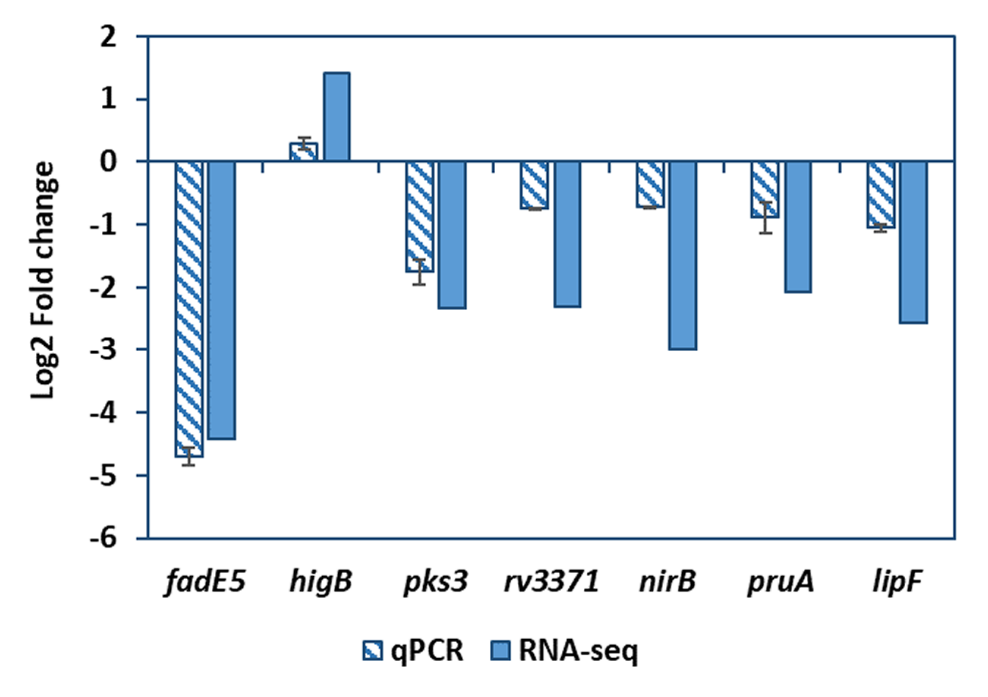


Figure S 2 The validation of seven RNA-sequencing differentially expressed genes using qPCR.

All fold-changes are relative to H54 in the exponential growth condition. For qPCR the expression values were normalized to rrs and fold-changes were computed based on 2^-ΔΔCT^ method. Bars with and without stripes indicate fold changes based on qPCR and RNA-seq respectively. Error bars indicate the standard deviation.


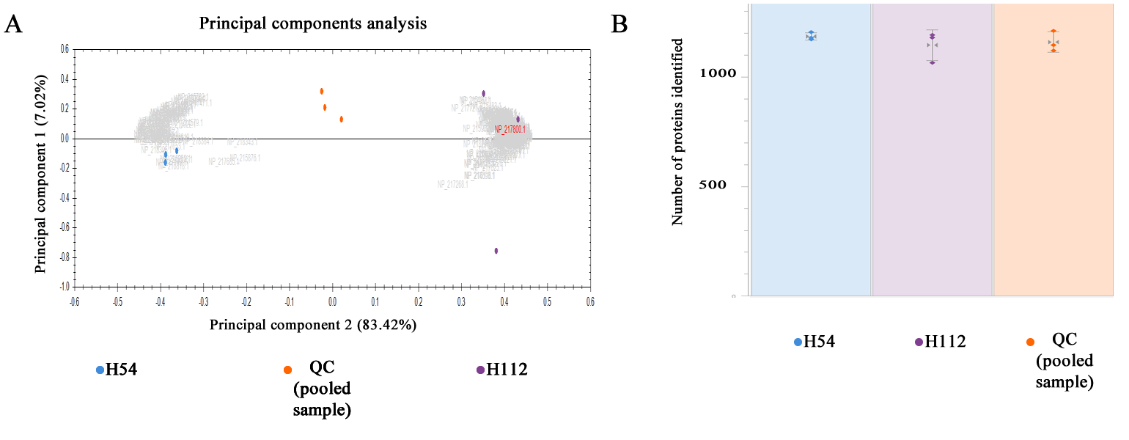


Figure S 3: Quality analysis of the proteomics data.

A) Principal component analysis on the protein abundance separates the samples H112 and H54 while pooled sample lies in the middle. B) The number of proteins identified in each sample were comparable across the samples as shown in the boxplot.

# Supplementary tables

Table S 1: Primer sequences used for qPCR validation of differential gene expression.

| **Target** | **Forward primer** | | | **Reverse primer** | | | **Product length (bp)** |
| --- | --- | --- | --- | --- | --- | --- | --- |
|  | **Sequence (5' -> 3')** | **Tm (°C)** | **GC%** | **Sequence (5' -> 3')** | **Tm (°C)** | **GC%** |  |
| *LipF* | AAGCAGGACCATCCCAACATC | 60.34 | 52.38 | CCGCCAAGGCATCGAATG | 59.28 | 61.11 | 67 |
| *pks3* | AGCAGCTGATGGCTTCGT | 59.33 | 55.56 | ATCCGGTAGCCGCTTCAG | 59.18 | 61.11 | 64 |
| *pruA* | TCTGCGAGCTGATCGACTTC | 59.9 | 55.00 | ATCGGCTGCTGCTCCAAAAT | 60.68 | 50.00 | 70 |
| *nirB* | TATACCGAAAGCTGGGACCG | 59.25 | 55.00 | TTAGTAGCAACCGAACCCGC | 60.39 | 55.00 | 82 |
| *rrs* | TCCCGGGCCTTGTACACA | 60.53 | 61.11 | CCACTGGCTTCGGGTGTTA | 59.63 | 57.89 | 62 |
| *HigB* | CGCTGATCGTGCTAATGCA | 58.7 | 52.63 | CCACGAATCGGTTTGATGCT | 58.91 | 50.00 | 70 |
| *Rv3371* | ACCAGCTCAAAACGGTTCTC | 58.41 | 50.00 | CAACACCTGGGTACATCGAG | 57.99 | 55.00 | 62 |
| *fadE5* | ATGTTGCCGGTAGTCAAGGG | 60.04 | 55.00 | GGGTTTGCAGGCTTTCAGTG | 59.97 | 55.00 | 70 |
